# Quality of mental health services and rights of people receiving treatment in inpatient services in Finland: a cross-sectional observational survey with the WHO QualityRights Tool Kit

**DOI:** 10.1186/s13033-021-00495-7

**Published:** 2021-08-28

**Authors:** Tella Lantta, Minna Anttila, Maritta Välimäki

**Affiliations:** 1 Department of Nursing Science, ICT-City, University of Turku, 20014 Turku, Finland; 2 Xiangya Nursing School, Central South University, 172 Tongzipo Road, Changsha, 410013 Hunan China

**Keywords:** World health organization, Psychiatric hospital, Quality, Service user, Family, Staff

## Abstract

**Background:**

This article aims to review the quality of mental health services and the rights of the people receiving treatment in inpatient hospital care in Finland using the World Health Organization’s QualityRights Tool Kit as a part of a randomized controlled trial VIOLIN. So far, reports on the QualityRights Tool Kit have mainly been from low- and middle-income countries or countries lacking resources for health services. Reports from countries with well-resourced health care systems, such as the Nordic countries, are still quite few.

**Methods:**

A cross-sectional observational survey was conducted on 13 closed inpatient psychiatric wards (acute, rehabilitation, forensic psychiatric) at eight hospitals in Finland. The data for the survey were gathered through a document review, observations, and group interviews among staff members, service users and family members. The STROBE checklist for cross-sectional studies was followed in the reporting.

**Results:**

Finnish mental health services are partially or fully achieving the standards set by the WHO QualityRights Tool Kit (final scores: 2.5–2.9 out of 3). The highest final score out of the five themes (2.9/3) was achieved under Freedom from torture or cruel, inhuman or degrading treatment or punishment and from exploitation, violence and abuse. The lowest final score out of the five themes (2.5/3) was achieved under the right to exercise legal capacity and the right to personal liberty and the security of person.

**Conclusions:**

According to the findings, Finnish mental health services appear to be of high quality. However, we have identified some gaps in quality, which we have addressed in a national randomized controlled trial VIOLIN. Improvements can be realized through shared decision making and relaying information to service users.

**Supplementary Information:**

The online version contains supplementary material available at 10.1186/s13033-021-00495-7.

## Background

Mental health is a human right to which every person is entitled. Every individual should have the right to high quality mental health services if care is needed [1]. However, there has been a rise in mental disorders worldwide, as well as in the challenges and barriers in the provision of scalable mental health services [2]. The right to the highest attainable standard of physical and mental health remains a distant goal for millions of people [3]. On a global level, the care available in mental health services is of poor quality, which hinders the recovery of patients. Many who have had access to care experience extensive restrictions of their basic human rights [4]. Therefore, the right to good mental health, mental health services and their assessment procedures should be a priority for every country and promoted in low- and middle-income countries (LMIC) as well as in high-income countries.

The WHO QualityRights, designed by WHO (the World Health Organization), aim to improve access to quality mental health and social services and promote the rights of people with mental health problems and other vulnerabilities. [4] To support this aim, a specific QualityRights Tool Kit has been developed in line with the Convention on the Rights of Persons with Disabilities (CRPD) to support countries in assessing and improving the quality of care and human rights conditions in mental health facilities [4, 5]. The Tool Kit is designed for use in LMIC and high-income countries, as it is an essential resource, not only for putting an end to past neglect and abuses but also for ensuring high-quality services in the future [5]. To date, the Tool Kit has been used for reviewing mental health facilities in many areas around the world, [6] including Egypt, [7] Tunisia, [8] Greece, [10] the Czech Republic, [11] Chile, [12, 13] and India. [14] These reports reveal a variety of deficits in mental health services, both in LMIC and in high-income countries, beginning with the poor physical state of the hospitals, [11] service users not being able to live independently in society, [9, 13] and a lack of preventive measures to avoid maltreatment and cruelty [13]. Authors have also concluded that informants may have been too afraid of punishment to share the true state of services, which has resulted in an overly optimistic picture of the quality of the care and fulfilment of human rights [7, 10]. Scarcity of resources was also evident in many countries [8, 11].

Currently, reports on the QualityRights Tool Kit are mainly from LMIC or countries lacking resources for their health services. There are very few reports for countries with well-resourced health care systems, such as the Nordic countries. It is therefore important to review the quality of the most developed heath care systems too, as when it comes to mental health, all countries are developing countries [15]. Finland, one of the five Nordic countries, enjoys a health care system ranked one of the highest in the world, being the 6th out of 195 countries according to the Global Burden of Diseases Study’s Healthcare Access and Quality (HAQ) Index [16]. The total mental health workforce per 1,00,000 people in Finland is the second highest in Europe [17]. The education level among the workforce in mental health services is high compared to other OECD countries (Organisation for Economic Cooperation and Development) [18]. Further, mental health services are low-cost or free for the users due to the budgeting system in the public sector, including that of the state, the municipalities and the Social Insurance Institution (Kela) [18]. In addition, the World Justice Project has ranked Finland as the third leading country out of 128 in protecting fundamental human rights worldwide [19].

On the other hand, deficits have also been detected in Finnish mental health services in terms of quality of care and human rights. Like many other Western European countries, Finland has followed the trend of deinstitutionalization by considerably reducing hospital-based care and beds since the 1990s [18]. Finnish psychiatric services are still, however, more hospital based than in other European countries [20]. There have also been questions of inefficient use of generous resources regarding productivity of mental health services when regions in Finland and Spain have been compared [21]. The burden of mental disorders on Finnish society is also high, with the disability-adjusted life years (DALY) (per 1,00,000 population) being 4092.98 [22]. For example, age-standardized DALY rates per 1,00,000 population for self-harm (males, 912.0 vs. 685.7) and depressive disorders (females, 933.7 vs. 812.0) are higher than average in Finland than in other Nordic countries [23]. Mental disorders are the most common reason for retirement with a disability pension, [24] and suicidality among young people is an ongoing worry [25]. According to Eurostat’s statistics, in 2016, the rate of suicide mortality among young people was higher in Finland than the average for EU countries [25]. In Finland, the crude death rate for people between 15 and 24 years was 15.54, whereas the corresponding average for EU countries was 5.81 [26]. In addition, the overall expenditure on mental health in Finland remains low compared to its burden on society [17, 18].

Regarding inpatient services, the use of coercion for service users has been debated during the last decades in Finnish mental health policy. From 2009 to 2015, national mental health plans strongly emphasized reducing the use of coercion in psychiatric care [27]. Despite a recent declining trend, the overall reduction in the use of coercive measures was small in Finnish psychiatric hospitals between 1995 and 2014 [28]. An international comparison among 22 countries recently showed that Finland has an above average rate of involuntary admission (151.4 vs. a median of 106.4 per 1,00,000 people) [29]. The European Committee for the Prevention of Torture and Inhuman or Degrading Treatment or Punishment (CPT) has also noted excessive use of coercion in Finnish psychiatric inpatient care and has stated that procedures related to involuntary admissions should be developed [30].

In this study, we aimed to review the quality of mental health services and rights of the people receiving treatment in inpatient hospital care in Finland using the WHO QualityRights Tool Kit [5]. This information was needed to set the groundwork for a subsequent randomized controlled trial (RCT), VIOLIN, which aimed to reduce the occurrence of seclusion events in psychiatric hospitals [31]. We chose the QualityRights Tool Kit because it provides a global and comprehensive assessment tool for psychiatric services to be used in different contexts [5]. Based on the knowledge gained, the strengths of the mental health services could be acknowledged, and especially the areas to be developed could be identified.

## Methods

### Aim and design

A cross-sectional observational survey of the quality of mental health services and the rights of the people receiving treatment in hospital care was conducted on 13 inpatient psychiatric wards at eight hospitals in Finland. Methods proposed by the WHO for using the QualityRights Tool Kit were adopted: the data for the survey were gathered through a document review, observations, and group interviews [5]. The STROBE (The Strengthening the Reporting of Observational studies in Epidemiology) checklist for cross-sectional studies was followed in the reporting of the study [32].

### Inclusion criteria and recruitment of the hospitals

Originally, we invited all 32 psychiatric hospitals in mainland Finland to join the RCT study with the following criteria: at least one ward for adult psychiatric care, open 24/7, coercive measures used on the ward, public and tax-funded, and no ongoing project on the ward with a similar type of study goal. Out of 32 possible hospitals, 15 hospitals with 28 wards were willing to participate in the RCT study. Wards were further randomly allocated into experimental and control wards on 11 May 2016, which resulted in 13 intervention wards. In this paper, we targeted the intervention study wards only.

### Recruitment of the study participants

The target groups of the study were health care staff, service users, and family members who were recruited using a convenience sampling method. Following WHO’s guideline, our aim was to recruit about 50% of the staff members and service users on each ward, and family members of around 25% of the service users on the ward during the data collection [5]. All staff members (psychiatrists, nurses, nurse managers, hospital cleaners, etc.) were eligible to participate, as long as they were on duty during the data collection period. Service users admitted on the ward and family members visiting them during the data collection period were eligible to join the study. All participants had to be adults (≥ 18 years old), able speak and read Finnish, and able to give informed consent. Participant groups were recruited via emails sent to the study wards, leaflets, and posters informing about the study and the times that the group interviews would be held.

### Data acquisition

The study material and the data collection procedures were pilot tested on one ward separately from this study (27–28 April 2016). The data based on document analysis, observations, and group interviews were collected between 25 May and 8 July 2016. Two researchers visited each of the 13 wards according to prescheduled visiting periods (Table 1). Each study ward collected preplanned material for the document analysis, which was shared with the researchers during the research visit (Additional file 1: Instructions for the document review). Group interviews were also organized with staff members, service users, and family members during the researchers’ visits to each of the 13 study wards. Due to the low number of family members present during the researchers’ visits to the wards, an additional invitation to participate in phone interviews with researchers was shared with family members. They were encouraged to call the researchers during a two-week period (27 June–8 July 2016).

**Table 1:** Location, ward characteristics and participants

Location	Ward characteristics				Participants		
Number and location of wards^a^	Type of ward	Number of health care staff (in total)	Patients/beds/average duration of treatment (days)^b^	Date of the visit	Staff members^c^	Service users	Family members
1—Helsinki-Uusimaa	Acute	N = 24.5	324/13/7	31/05/2016	8/16 50%	2	1^d^
		Psychiatrist, n = 3					
		Nurses, n = 15 (RN), n = 6 (PN)					
		Psychologist, n = 0					
		Social worker, n = 0.5					
2—Northern and Eastern Finland	Acute/rehabilitation	N = 34	280/24/27	20/06/2016	12/20 60%	4	0
		Psychiatrist, n = 1					
		Nurses, n = 16 (RN), n = 17 (PN)					
		Psychologist, n = 0					
		Social worker, n = 0					
3—Western Finland	Acute/rehabilitation	N = 48	469/32/17	07/06/2016	14/28 50%	4	1^d^
		Psychiatrist, n = 3					
		Nurses, n = 26 (RN), n = 14 (PN)					
		Psychologist, n = 2					
		Social worker, n = 3					
4—Western Finland	Acute	N = 35	666/16/4	01/06/2016	13/23 57%	8	0
		Psychiatrist, n = 3					
		Nurses, n = 25 (RN), n = 5 (PN)					
		Psychologist, n = 1					
		Social worker, n = 1					
5—Northern and Eastern Finland	Rehabilitation/forensic	N = 22	78/16/41	08/06/2016	10/14 71%	2	1
		Psychiatrist, n = 1					
		Nurses, n = 9 (RN), n = 10 (PN)					
		Psychologist, n = 1					
		Social worker, n = 1					
6—Northern and Eastern Finland	Acute/rehabilitation	N = 37	237/30/10	09/06/2016	13/22 59%	3	1
		Psychiatrist, n = 1					
		Nurses, n = 19 (RN), n = 15 (PN)					
		Psychologist, n = 1					
		Social worker, n = 1					
7—Northern and Eastern Finland	Acute	N = 27	442/12/9	25/05–26/05/2016	8/14 57%	4	0
		Psychiatrist, n = 3					
		Nurses, n = 13 (RN), n = 9 (PN)					
		Psychologist, n = 1					
		Social worker, n = 1					
8—Northern and Eastern Finland	Acute	N = 23	259/12/9	25/05/2016	9/15 60%	5	0
		Psychiatrist, n = 2					
		Nurses, n = 12 (RN), n = 8 (PN)					
		Psychologist, n = 0					
		Social worker, n = 1					
9—Southern Finland	Acute	N = 26	388/23/9	16/06/2016	6/19 32%	0	1^d^
		Psychiatrist, n = 2					
		Nurses, n = 20 (RN), n = 2 (PN)					
		Psychologist, n = 1					
		Social worker, n = 1					
10—Southern Finland	Rehabilitation	N = 22	93/20/53	14/06–17/06/2016	9/11 82%	4	0
		Psychiatrist, n = 1					
		Nurses, n = 12 (RN), n = 6 (PN)					
		Psychologist, n = 1					
		Social worker, n = 2					
11—Southern Finland	Acute	N = 26	412/23/9	14/06/2016	11/20 55%	5	0
		Psychiatrist, n = 2					
		Nurses, n = 20 (RN), n = 2 (PN)					
		Psychologist, n = 1					
		Social worker, n = 1					
12—Southern Finland	Rehabilitation/forensic	N = 23	42/20/50	02/06/2016	12/16 75%	2	0
		Psychiatrist, n = 1					
		Nurses, n = 17 (RN), n = 3 (PN)					
		Psychologist, n = 1					
		Social worker, n = 1					
13—Southern Finland	Rehabilitation	N = 22	67/16/65	15/06/2016	5/10 50%	2	0
		Psychiatrist, n = 1					
		Nurses, n = 15 (RN), n = 4 (PN)					
		Psychologist, n = 1					
		Social worker, n = 1					

*RN* registered nurse; *PN* practical nurse

^a^According to regional classification NUTS 2016 (https://www.stat.fi/meta/luokitukset/nuts/002-2018/fi1_en.html)

^b^Number of patients who have had treatment during one year period/number of patient beds on the ward, including extra beds/approximate duration of the treatment on the ward as days

^c^All staff members (including nurse managers, and hospital cleaners etc.) were eligible to participate. Numbers are presented as how many participated in the interviews out of total number of staff on duty during the time of visit

^d^Interviews were conducted via phone

As part of the VIOLIN study, at the beginning of 2017, we collected the following information from the study wards: the number of health care staff, the number of service users and beds, and the duration of treatment (days) in 2016 (Table 1).

#### The WHO QualityRights Tool Kit

The WHO QualityRights Tool Kit (2012) was selected as the data collection tool as it has been found to be suitable for use in countries with different income levels [3]. More specifically, the Review of documents and Observation Tool was used to provide guidance on reviewing relevant documentation and observing a facility [5]. To supplement this document and observation review, the Interview Tool was used to give guidance in conducting group interviews [5].

The WHO QualityRights Tool Kit contains five main themes: (1) the right to an adequate standard of living, (2) the right to enjoyment of the highest attainable standard of physical and mental health, (3) the right to exercise legal capacity and the right to personal liberty and the security of person, (4) freedom from torture or cruel, inhuman or degrading treatment or punishment and from exploitation, violence and abuse, and (5) the right to live independently and be included in the community [5]. Each of the themes has 4–7 standards to evaluate based on the document review, observations of the researchers, and/or interviews (Table 2).

**Table 2:** QualityRights Tool Kit review

Themes and standards	Wards													
	1	2	3	4	5	6	7	8	9	10	11	12	13	Total (mean, SD)
Theme 1 the right to an adequate standard of living														
1.1: the building is in good physical condition	2	2	2	3	2	2	2	3	3	3	2	3	3	2.5 (0, 5)
1.2: the sleeping conditions of service users are comfortable and allow sufficient privacy	2	2	2	3	2	3	2	3	2	3	2	3	3	2.5
1.3: the facility meets hygiene and sanitary requirements	2	2	3	3	2	3	3	3	3	3	3	3	3	2.8
1.4: service users are given food, safe drinking-water and clothing that meet their needs and preferences	3	3	3	2	3	3	3	3	3	3	3	3	3	2.9
1.5: service users can communicate freely, and their right to privacy is respected	2	3	2	3	3	3	3	3	3	3	2	2	2	2.6
1.6: the facility provides a welcoming, comfortable, stimulating environment conducive to active participation and interaction	2	2	2	2	3	2	2	2	2	3	3	3	3	2.4
1.7: service users enjoy a fulfilling social and personal life and remain engaged in community life and activities	2	2	2	2	3	3	2	2	2	3	3	3	3	2.5
Final score	2	2	2	2	3	3	2	3	3	3	3	3	3	2.6
Theme 2 the right to enjoyment of the highest attainable standard of physical and mental health														
2.1: facilities are available to everyone who requires treatment and support	3	3	3	3	3	3	3	3	3	3	3	3	3	3.0
2.2: the facility has skilled staff and provides good quality mental health services	2	2	3	2	2	2	2	2	3	3	3	3	3	2.5
2.3: treatment, psychosocial rehabilitation and links to support networks and other service are elements of a service user-driven recovery plan and contribute to a service user's ability to live independently in the community	3	2	3	2	2	3	3	2	2	3	2	3	3	2.5
2.4: psychotropic medication is available, affordable and used appropriately	3	2	3	2	3	2	2	2	3	3	2	3	3	2.5
2.5: adequate services are available for general and reproductive health	2	2	3	3	3	3	2	2	3	3	3	3	3	2.7
Final score	3	2	3	2	3	3	2	2	3	3	3	3	3	2.7
Theme 3 the right to exercise legal capacity and the right to personal liberty and the security of person														
3.1: service users' preferences regarding the place and form of treatment are always the priority	3	2	3	2	3	3	2	2	2	2	2	3	2	2.4
3.2: procedures and safeguards are in place to prevent detention and treatment without free and informed consent	3	2	2	3	2	2	3	2	3	3	2	2	2	2.4
3.3: service users can exercise their legal capacity and are given the support they may require to exercise their legal capacity	2	2	2	2	3	3	2	2	2	3	3	3	3	2.5
3.4: service users have the right to confidentiality and access to their personal health information	3	3	2	2	2	3	3	3	3	3	3	2	3	2.7
Final score	3	2	2	2	2	3	3	2	3	3	3	2	2	2.5
Theme 4 freedom from torture or cruel, inhuman or degrading treatment or punishment and from exploitation, violence and abuse														
4.1: service users have the right to be free from verbal, mental, physical and sexual abuse and physical and emotional neglect	2	2	3	3	3	3	3	2	3	3	3	2	3	2.7
4.2: alternative methods are used in place of seclusion and restraint as means of de-escalating potential crises	2	2	2	2	2	2	2	2	2	2	2	3	2	2.1
4.3: electroconvulsive therapy, psychosurgery and other medical procedures that may have permanent or irreversible effects, whether performed at the facility or referred to another facility, must not be abused and can be administered only with the free and informed consent of the person	3	3	3	3	3	3	3	3	3	3	3	3	3	3.0
4.4: no service user is subject to medical or scientific experimentation without his or her informed consent	3	3	3	3	3	3	3	3	3	3	3	3	3	3.0
4.5: safeguards are in place to prevent torture or cruel, inhuman or degrading treatment and other forms of illtreatment and abuse	3	3	3	3	3	3	3	3	3	3	3	3	3	3.0
Final score	3	2	3	3	3	3	3	3	3	3	3	3	3	2.9
Theme 5 the right to live independently and be included in the community														
5.1: service users are supported in gaining access to a place to live and have the financial resources necessary to live in the community	3	3	3	3	3	2	3	3	3	3	3	3	3	2.9
5.2: service users can access education and employment opportunities	3	3	3	2	2	3	3	3	2	3	3	3	3	2.8
5.3: the right of service users to participate in political and public life and to exercise freedom of association is supported	3	2	3	3	3	3	2	2	2	3	3	3	3	2.7
5.4: service users are supported in taking part in social, cultural, religious and leisure activities	2	3	2	2	2	3	2	2	3	3	3	2	3	2.5
Final score	3	2	3	2	3	3	3	2	2	3	3	3	3	2.7

During the researchers’ visits, the document review was done by two researchers going through the pre-collected material with the Review of documents and observation Tool [5]. This same tool was used for observations of the researchers. Interviews for each participant group were organized on each study ward at prescheduled times with the Interview Tool [5]. Separate group discussions were held with the various stakeholder groups—staff, service users, and families.

Each standard included in the themes was scored as follows: 3 points meant the standard was being fully achieved, 2 points meant standard was being partially achieved, 1 point meant that there was no evidence that the standard had been initiated or that any attempts had been made to fulfil the criteria, and 0 points meant that the standard was not applicable in that environment. A final score for each theme was given based on a scoring average of standards included for that particular theme.

Two WHO (2012) standards were left out during our data collection for the following ethical and practical reasons. First, Standard 2.32, “Treatment, psychosocial rehabilitation and links to support networks and other services are elements of a service user-driven recovery plan,” was left out as we were not able to review all medical records due to a lack of ethical approval. Second, Standard 2.4, “Psychotropic medication is available, affordable and used appropriately,” was excluded due to the lack of ethical approval to review patient medical records and because it was not plausible to recruit a pharmacist to the study team.

### Data analysis

Descriptive statistics were used to illustrate the participating hospital wards. Each standard in the QualityRights Tool Kit scoring sheet (25 in total) was evaluated by two researchers with a master’s or doctoral degree in health sciences, a professional higher degree in nursing or the equivalent, and experience in psychiatric inpatient care as a nurse and/or researcher. Both researchers scored the material independently based on the document review, observations, and interviews. Interviews were a complementary element of this review. If a standard was fully met based on the documents and observations, and staff members verified this in their interviews, but service users and family members described the opposite, the specific standard was scored as “partially achieved.” If there were any differences in the evaluations, the study material was reviewed again until a consensus was reached.

Interview data from staff members, service users and family members’ interviews are included in the Results section as participant quotations to enrich the study findings. Directed coding techniques [33] were used to search for relevant quotations from the taped interviews that matched the standards in the scoring sheets. Quotations were selected based on their suitability to illustrate the study findings and their representativeness of different study wards. Tapes from the interviews were first listened through, and second, keeping in mind the standards of the QualityRights Tool Kit, quotations were transcribed from the tapes to a Word document.

## Results

Altogether, 13 visits (during 1 or 2 days) to the wards were prescheduled and realized during the data collection period. Most wards were located in Northern and Eastern Finland (f  =  5, 38%) and Southern Finland (f  =  5, 38%). All wards were closed. Almost half (f  =  6, 46%) were acute inpatient wards for adults, and the rest were rehabilitation and forensic psychiatric wards. On average 289 service users [standard deviation (SD) 187.3] received treatment on the wards annually, and there were approximately 20 beds (SD 6.4). The average length of treatment periods was 24 days (SD 21.10).

A total of 130 staff members, 45 service users and five family members (three through individual telephone interviews) participated in reviewing the quality of mental health services and the rights of the people receiving treatment in inpatient services. Table 1 offers an overview of the interview locations, ward characteristics and the participants in the group interviews.

Results are presented here with the final scores of the themes, as theme standards with the lowest and highest scores (Table 2), and with participant quotes extracted from the taped interviews.

### Theme 1: the right to an adequate standard of living

The right to an adequate standard of living in Finland was partially achieved, with a final score of 2.6/3 based on seven standards. The standard dealing with the ward environment received the lowest score (2.4), whereas the standard related to food, safe drinking-water and clothing was almost fully achieved (2.9).

Ward spaces are just too small. There are too few meeting rooms. Rooms for service users are for two persons. If families come to visit, there is not much privacy.

[Staff, identifier (ID) 1]

This ward environment is bright and beautiful. I had expected gloomier.

(Family member, ID 3)

Here you are never left with hunger.

(Patient, ID 19)

### Theme 2: the right to enjoyment of the highest attainable standard of physical and mental health

The right to health was partially achieved with a final score of 2.7/3. Three standards scored below this average with a score of 2.5 (standards related to skilled staff, good-quality health services; treatment, psychosocial rehabilitation, and links to support networks; availability of and use of psychotropic mediation). The standard about the availability of the facilities to anyone requiring treatment and support was fully achieved (3/3) on every ward.

I personally would want more occupational therapeutic and physiotherapeutic services, maybe even a dietician. These services are not provided here much.

(Staff, ID 35)

It would be good if you would get more information about these medications. Do they make you swell, and what other side effects might there be?

(Patient, ID 37)

I have not been told anything except that a doctor said, “See you tomorrow.” I have been kept in the dark. Blood samples have been taken, but I have not been told the results or why the samples were taken.

(Patient, ID 38)

Here, (service users) are taken care of from head to toe.

(Staff, ID 102)

### Theme 3: the right to exercise legal capacity and the right to personal liberty and the security of person

The theme covering the right to exercise legal capacity, to personal liberty and to security was partially achieved, with the lowest final score out of the five themes, 2.5/3. Two standards scored below the final score, 2.4 (the standard related to the respect of service users’ preferences regarding the place and form of treatment, and the standard related to safeguards and procedures preventing detention and treatment without free and informed consent), while the standard related to the right to confidentiality and access to personal health information achieved the highest score, 2.7.

I have heard about Kanta.fi (to get access to electronic personal health information), but I don’t think one can see everything they want to see there. I have not visited there. Staff have not informed me about this.

(Patient, ID 1)

It (information about patient rights) is not given on a regular basis, but given if asked. Today, a patient came to say that they wanted to file a complaint. Then we told them how to do it. So yeah, we are taking care of these kinds of things.

(Staff, ID 125)

### Theme 4: freedom from torture or cruel, inhuman or degrading treatment or punishment and from exploitation, violence and abuse

Theme 4, involving freedom from torture or degrading treatment, was almost fully achieved and scored highest out of the five themes with 2.9/3. The standard related to the use of alternative methods in place of seclusion and restraint achieved a score of 2.1, which was the lowest out of the 25 standards evaluated. However, three standards out of five achieved a full score (3/3): the standard related to the non-abuse of electroconvulsive therapy, the standard about scientific experimentation, and the standard about safeguards in place to prevent ill-treatment.

Human beings, after all, do not deserve seclusion. There is not even a toilet, but you have to pee in the corners. You just have a blanket around you. You have a pretty helpless and naked feeling afterwards.

(Patient, ID 1)

We have not made any real agreements about treatment (with the service users), like “if I’m feeling this way, treat me like this.” If there are 20 service users on the ward and each of them would like to receive different care, we would get all confused. So we have to go with our own ways of providing care.

(Staff, ID 88)

### Theme 5: the right to live independently and be included in the community

The right to independent living and to being included in one’s community was partially achieved with a final score of 2.7/3. The lowest score in this theme was given to the standard about support in taking part in different community free-time activities, 2.5. One standard scored above the final score: the standard about gaining access to a place to live and having the financial resources necessary to live in the community, 2.9.

I need to find accommodation. I did not get a positive reaction from the social worker. I should have done it myself (sought accommodation). I don’t know why the social worker did not support my efforts or that we could not start searching together.

(Patient, ID 21)

If we are thinking about supported accommodation, usually a patient goes to these places with a nurse. That is before the official visit and negotiation. The patient and the nurse visit the webpages (of the accommodation place) together.

(Staff, ID 114)

## Discussion

In this paper, we present findings from a study reviewing the quality of mental health services and the rights of people receiving treatment in inpatient services in Finland. According to our knowledge, this is the first paper reporting the use of the WHO QualityRights Tool Kit in a high-income Nordic country. The analysis shows that Finnish mental health services are partially or fully achieving all the standards set by the WHO QualityRights Tool Kit (the final scores 2.5–2.9 out of 3) [5]. The results of this study confirm the high quality of Finnish mental health services but also reveal areas where further improvements are needed. This shows that, when it comes to care of the most vulnerable service user group, it is not enough to just assume that the quality of care is automatically ensured in high-income countries. In this section, we discuss key strengths of the Finnish mental health system, but also standards that, in light of our document review, observations, and/or interviews, have not been fully achieved.

The highest final score out of the five themes (2.9/3) was achieved under the theme Freedom from torture, cruel, inhuman or degrading treatment or punishment and from exploitation, violence and abuse. This result is opposite to what Minoletti et al. [13] found in their study, which revealed a lack of preventive measures to avoid maltreatment and cruelty in South American mental health facilities. Our results can be considered obvious as Finland is one of the leading countries in the world in protecting the fundamental human rights of all citizens. [19] On the other hand, the standard related to the use of alternative methods in place of seclusion and restraint received the lowest score (2.1) out of the 25 standards evaluated. Coercive methods used in treatment can be verified in the study results of Välimäki et al. [28] which show that the overall reduction of coercive measures in Finnish psychiatric hospitals has been found to be small during the last two decades.

However, coercive methods in care should not be used only because there is a lack of available alternative methods for seclusion and restraint. For example, in their systematic mapping review, Baker et al. [34] found 105 interventions to reduce restrictive practices in adult psychiatric inpatient settings. Most of them were multicomponent interventions tested in non-randomized designs. Only six randomized controlled trials were found. Across these studies, interventions were poorly described, fidelity was not measured, and they lacked a theoretical basis. Thus, implementing such interventions into clinical practice can be very difficult. Another umbrella review highlighted evidence of the benefits of staff training, shared decision-making and integrated care interventions in reducing coercive treatment in psychiatric services [35]. Fortunately, after our data collection, more evidence-based, effective alternatives for seclusion and restraint have been put in place in Finland. Alternative interventions based on, for instance, tested interventions such as the Safewards and the Six Core Strategies, are now widely spread across Finland [36]. However, there are large deviations between wards in terms of adopting alternatives. There is still room for further development in inpatient psychiatric services as the total mental health workforce is high in Finland, [17] and no scarcity of resources was found in our study, unlike in many other countries [8, 11].

Standards about availability of facilities to anyone who requires treatment and support, non-abuse of electroconvulsive therapy, scientific experimentation, and safeguards being in place to prevent ill-treatment all received a score of 3/3, indicating that they are being fully achieved. In Finland, several acts, such as the Mental Health Act (1116/1990), [37] the Health Care Act (1326/2010), [38] and the Act on the Status and Rights of Patients (785/1992), [39] protect service users and create prerequisites for quality of treatment. There are regulations about patient information registers and handling of patient data (Health Care Act 1326/2010), [38] and about treating illness with mutual understanding (Act on the Status and Rights of Patients 785/1992) [39]. However, mental health care legislation in Finland is rather dated. One could  question if the Mental Health Act from 1990 is still valid and up to date in the 2020s. Putkonen and Völlm [40] concluded that the medical orientation of the Finnish mental health legislation, over individuals’ civil liberties, may be one reason for the high rates of involuntary care. Välimäki et al. [41] have described the Finnish approach to involuntary treatment as paternalistic. Also, the option for compulsory outpatient treatment is missing from Finnish national legislation, an option that could be beneficial for forensic psychiatric patients [40].

The lowest final score out of five themes (2.5/3) was received by the right to exercise legal capacity, personal liberty and the security of person. Our study wards were acute, rehabilitation, and forensic psychiatric wards. They were closed wards for service users with severe mental illnesses in an acute state (F20 Schizophrenia being the most common diagnosis) [42]. Service users in acute and forensic psychiatric wards are often hospitalized against their will, and therefore, our results may be biased towards the views of people treated against their will in a restrictive environment [29]. In Finland, a person can be admitted to involuntary care if they are diagnosed as mentally ill. This is done in cases when the person’s health or safety or the health or safety of others would be endangered if treatment was not administered, and if all other mental health services would be inapplicable or inadequate [Mental Health Act (1116/1990)] [37]. The forensic psychiatric wards in our study dealt with service users whose treatment could be particularly dangerous or difficult [Mental Health Act (1116/1990)] [37]. Participants in our study may therefore have had strong feelings about being treated against their will and being kept in a restricted environment, which may explain part of the results. Therefore, keeping in mind that current Finnish service approaches emphasize community care and outpatient services (Mental Health Finland 2021), the results cannot be generalized to all mental health services in Finland [43].

In previous studies, authors have suggested that informants may have provided an overly optimistic picture of the quality of care and fulfilment of human rights [7, 10]. In Finland, this may not be the case as all people have the fundamental right to express their opinion (The Constitution of Finland 731/1999), [44] and Finland ranks second in the World freedom of expression rankings 2019/20 [45]. Nevertheless, it has been stated that the right of service users to exercise their legal capacity may be difficult in Finland due to the complexity of the complaint process [46]. In our study, service users mentioned that they had not been properly informed about their rights to complain or how to contact a patient ombudsman. Thus, we may question if wards are doing enough to promote service users’ civil and legal rights while they are hospitalized. Already over a decade ago, Kuosmanen et al. [47] suggested that professionals working in the field of mental health need education on how to support service users in exercising their legal capacity. Also at the international level, there is an urgent need for effective interventions to strengthen service user complaint systems [48]. This study suggests that Finnish mental health policy makers should further discuss what actions should be taken to improve the situation. The results also suggest the need for a national review in psychiatric facilities, in line with suggestions from the CPT [30]. Structured interventions to aid the realization of legal rights, like OPeNS, [49] may be needed to support service users, especially when involuntary admitted to psychiatric care, to adequately fulfil WHO’s standards.

There is also room for improvement in service users’ preferences regarding treatment, informed consent, and facilities (standard score 2.4/3). A recent interview study among Finnish clinicians working in psychiatric care concluded that there seems to be a shift from paternalistic treatment decisions towards shared decision making (SDM) and patient-centered care [50]. From the viewpoints of service users and their families, these improvements are still modest [51, 52]. Positive development could be promoted by implementing tested approaches to aid SDM, such as SDM-Plus, [53] in Finnish psychiatric facilities. SDM-Plus provides training for service users, in addition to professionals. This could be useful in Finland as patients may not be used to taking part in their care decisions, due to, for instance, a lack of decisional capacity or the feeling of powerlessness during involuntary treatment [54]. Further, the role and process of psychiatric advance statements and other crisis planning should be promoted and made clearer in Finland to better fulfill the treatment preferences of service users. This would also help reduce the number of compulsory admissions [55, 56].

Our study revealed regional differences in facilities regarding resources that inpatient units have. In terms of physical environments, not all of the wards were able to provide a “welcoming, comfortable, stimulating environment conducive to active participation and interaction” (Table 2). Some wards we visited operated in transitory premises, designed for the care of physical illnesses, but not meant for people recovering from mental health crises. As it has already been stated, psychiatric facilities may not always look and function like places for care and recovery [57]. Fortunately, new psychiatric hospitals have been opened since our data collection took place, and several construction projects are underway in Finland.

In terms of human resources, the number of health care staff ranged from 22 to 48, varying from several psychiatrists, psychologists, and social workers on the ward to just one or none at all. The number of service users on the wards ranged from 42 to 666 annually, and the number of beds, from 12 to 32, meaning that wards varied from those who provided care for many service users with minimal bed capacity (e.g., acute care) to those who had fewer service users and more beds (e.g., rehabilitation). A study conducted in the Finnish capital region confirmed WHO’s information about well-resourced mental health services, but this descriptive information collected in our study highlights the need for further investigation of how much variation exists in terms of available resources within the country [17, 21].

This study has limitations. The study results are only generalizable to 13 inpatient wards in Finland. Although all of the main geographical regions in Finland were represented in the study, hospitals in two regions might be overrepresented. Our original inclusion criteria (closed wards with psychiatric beds and specific facilities for patient seclusion) might have determined that 28 hospitals out of 32 were deemed eligible, of which 13 hospitals refused to participate. The main reason for refusal was that other similar research projects were ongoing or about to start. We did not collect demographic information from the participants, so we cannot state if some groups (age, gender) were underrepresented in the interviews or if those who participated differed from those who did not consent to participate. However, we know that all participants were adults (18 years old or older), and all study wards were mixed-gender wards. Based on the inclusion criteria, all participants were able to speak and read Finnish. The evaluations done in this study left out a few standards, impairing the quality of the evaluation. Moreover, the evaluations are based on the reviews of two researchers, material pre-collected on the wards, and four scores for each standard, all elements that could have potentially reduced the validity of the results. A more versatile composition of the research groups, for example, with experienced experts and pharmacologists, could have improved the thoroughness of the evaluations. Further, our study did not achieve the sample size of family members that it aimed for. At some sites, the number of service users was small, and on one ward, we were unable to recruit any service user participants at all. These recruitment issues hinder the study validity, as statements made by staff members cannot be triangulated with findings from a sufficient number of service users and family members.

## Conclusions

To summarize, the quality of Finnish psychiatric inpatient services is at a high level, based on our review. However, the analysis reveals areas where improvements are needed, such as the rights to exercise legal capacity, personal liberty, and the security of person, which are fundamental rights of all human beings. Even though torture and degrading treatment does not exist in psychiatric facilities in Finland, alternative methods to seclusion and restraint are still needed. In our VIOLIN trial, we were able to use this review as groundwork for an evidence-based educational intervention. Future steps in Finland could include implementing the WHO QualityRights training program in all psychiatric hospitals [58]. As proposed by Duffy and Kelly in Ireland, [59] this training could reduce coercive practices, which we also found problematic in our study. Improvement in service users’ preferences regarding treatment, informed consent and facilities could be realized through shared decision making and keeping service users informed. A nationwide review using the WHO QualityRights Tool Kit, in different kinds of facilities, including outpatient units, would be needed to gain a more comprehensive and comparable picture of the Finnish mental health care system.

## Supplementary Information

**Additional file 1: **Instructions for the document review.

## Data Availability

The datasets used and/or analyzed during the current study are available from the corresponding author on reasonable request.

## Abbreviations

CPT: The European Committee for the Prevention of Torture and Inhuman or Degrading Treatment or Punishment
CRPD: Convention on the Rights of Persons with Disabilities
DALY: Disability-adjusted life years
HAQ: Healthcare access and quality
ID: Identifier
Kela: Social Insurance Institution
LMIC: Low- and middle-income countries
OECD: Organisation for Economic Cooperation and Development
RCT: Randomized controlled trial
SD: Standard deviation
STROBE: The Strengthening the reporting of observational studies in epidemiology
WHO: World Health Organization
