# Supplementary material for: Quality of mental health services and rights of people receiving treatment in inpatient services in Finland: a cross-sectional observational survey with the WHO QualityRights Tool Kit
Source: Int J Ment Health Syst. 2021 Aug 28;15:70. doi: 10.1186/s13033-021-00495-7 (PMC8399820; doi:10.1186/s13033-021-00495-7)
Supplement: Supplementary file 1 — Additional file 1: Instructions for the document review. [file 13033_2021_495_MOESM1_ESM.docx]

Appendix 1. Instructions for the document review

**WHO QualityRights Tool Kit document review (WHO 2012)**

*A review of the wards and organizational documents*

Before each visit to a ward, the study contact persons received a document list (in Finnish) from the research team. They were asked to gather all the relevant documents (e.g., instructions, house rules, orders, and leaflets) related to following themes:

- fire and health and safety regulations
- sleeping conditions in the facility
- cleaning procedures on the ward
- service users’ access to toilet and bathing facilities
- content and amount of food given to service users, including weekly or monthly menus
- patient clothing
- service users’ access to means of communication and to communication in their preferred language
- visitors
- service users’ movements around the facility
- policies for the provisions of equipment and space to be used by service users for leisure activities
- interaction among service users
- service users’ attendance at personal social activities, such as funerals
- sharing of information by staff with service users and support for 1) their access to housing and financial resources, 2) education and employment opportunities, 3) political activities and participation in political, religious, social and disability organizations, 4) social, cultural, religious and leisure activities
- admission criteria for service users
- a referral policy
- mandatory continuing training of staff
- policy to let service users express their opinions on service provisions and improvements
- general and reproductive health services
- acknowledging service users’ preferences in all matters pertaining to where they wish to receive treatment
- promotion of service users’ preferences regarding treatment and recovery options
- directing staff to provide information about admission and/or treatment to all service users and obtain their consent
- advance directives/statements
- documenting and reporting the admission or treatment of a service user against their will
- informing service users of their right to appeal their admission or treatment and the procedures for doing so, including how to access appeals procedures and legal representation if needed or desired
- providing information to service users and discussing their assessment, treatment and recovery options
- assisting service users in accessing support for decision making
- keeping service user information confidential and giving service users access to their personal information, including possibilities to comment on them
- guidance on prevention, reporting and dealing with service users neglect and abuse during the hospital treatment
- use of seclusion and restraint (use, documenting, reporting)
- alternative methods to use of seclusion and restraint
- policy regarding electroconvulsive therapy (ECT)
- policy on psychosurgery and other invasive or irreversible treatments
- policy that addresses abortions and sterilizations
- policy on medical and other forms of experimentation
- visits to the facility by independent monitoring authorities
